# Supplementary material for: Resource and seasonality drive interspecific variability in simulations from a dynamic energy budget model
Source: Conserv Physiol. 2023 Mar 30;11(1):coad013. doi: 10.1093/conphys/coad013 (PMC10064112; doi:10.1093/conphys/coad013)
Supplement: Web_Material_coad013 [file web_material_coad013.zip › Marino_et-al_2021_ConsPhys_Supplementary Materials.pdf]

Supplementary Material for "Resource and seasonality drive interspecific variability in simulations from a Dynamic Energy Budget model"

Joany Mariño[a,*], Suzanne C. Dufour[a], Amy Hurford[a,b], Charlotte Récapet[c]

[a] *Department of Biology, Memorial University of Newfoundland. St. John's A1B 3X9, Canada*
[b] *Department of Mathematics and Statistics, Memorial University of Newfoundland. St. John's A1C 5S7, Canada*
[c] *UMR ECOBIOP, INRAE-Université Pau Pays de l'Adour, 64310 St Pée sur Nivelle, France*

1. Parameter space validation

There are 14 species in the AmP collection (on March 2020) modelled with the DEB-std model with parameter values that lie within our parameter space for $\dot{v}$, $\{\dot{p}_{Am}\}$, and $[\dot{p}_M]$ (Table 1).

*Corresponding author
Email address: jmarinocoron@mun.ca (Joany Mariño)

Table 1. Species in the AmP collection with parameter values within our parameter space for $\dot{v}$, $\{\dot{p}_{Am}\}$, and $[\dot{p}_M]$. The coefficient of variation (c_v) summarizes the parameter variation across species. See Table 2 in the main file for parameter notation and units.

Species	Common name	κ	$\{\dot{p}_{Am}\}$	$\dot{v}$	$[\dot{p}_M]$	$[E_G]$	E_H^b	E_H^p
Acanthiza chrysorrhoa	Yellow-rumped thornbill	0.996	4724.4	0.50	1992.8	7328.6	54.9	10830
Acanthiza inornata	Western thornbill	0.995	4005.6	0.47	1881.1	7310.6	76.9	7243
Anthus spinoletta	Water pipit	0.997	3286.2	0.27	1670.3	7316.1	36.6	1974
Calidris minuta	Little stint	0.932	3898.4	0.19	1973.6	7338.4	1354.0	387000
Emberiza calandra	Corn bunting	0.915	4797.9	0.41	1949.0	7307.3	1758.0	999500
Ficedula hypoleuca	European pied flycatcher	0.822	3350.8	0.22	1642.7	7310.7	1389.0	823600
Geospiza fortis	Medium ground finch	0.968	3146.2	0.32	1644.7	7316.3	325.9	165000
Hemiphaga novaeseelandiae	New Zealand pigeon	0.990	6900.6	0.17	1605.2	7316.0	570.1	616800
Molothrus bonariensis	Shiny cowbird	0.961	4122.9	0.21	1740.8	7332.9	766.0	277700
Motacilla aguimp	African wagtail	0.999	3804.9	0.45	1670.3	7290.4	13.4	1957
Motacilla citreola	Citrine wagtail	0.999	3204.1	0.31	1670.3	7312.9	6.4	860
Motacilla clara	Mountain wagtail	0.998	3109.4	0.22	1670.3	7330.5	15.1	1355
Sterna paradisaea	Arctic tern	0.991	5604.5	0.45	1681.2	7359.8	691.8	230000
Turdus migratorius	American robin	0.981	4940.2	0.32	1734.4	7306.1	440.3	312100
	Mean	0.967	4206.9	0.32	1751.9	7319.8	535.6	273994
	c_v	0.05	0.26	0.36	0.08	0.00	1.10	1.20

2. Model validation

At constant food levels, the relative differences between our simulations for reserve density and the analytical solutions (equation 5 in the main text) are close to zero (Figure 1). These small differences at each of the five constant resource levels we evaluated show the consistency of our simulations.

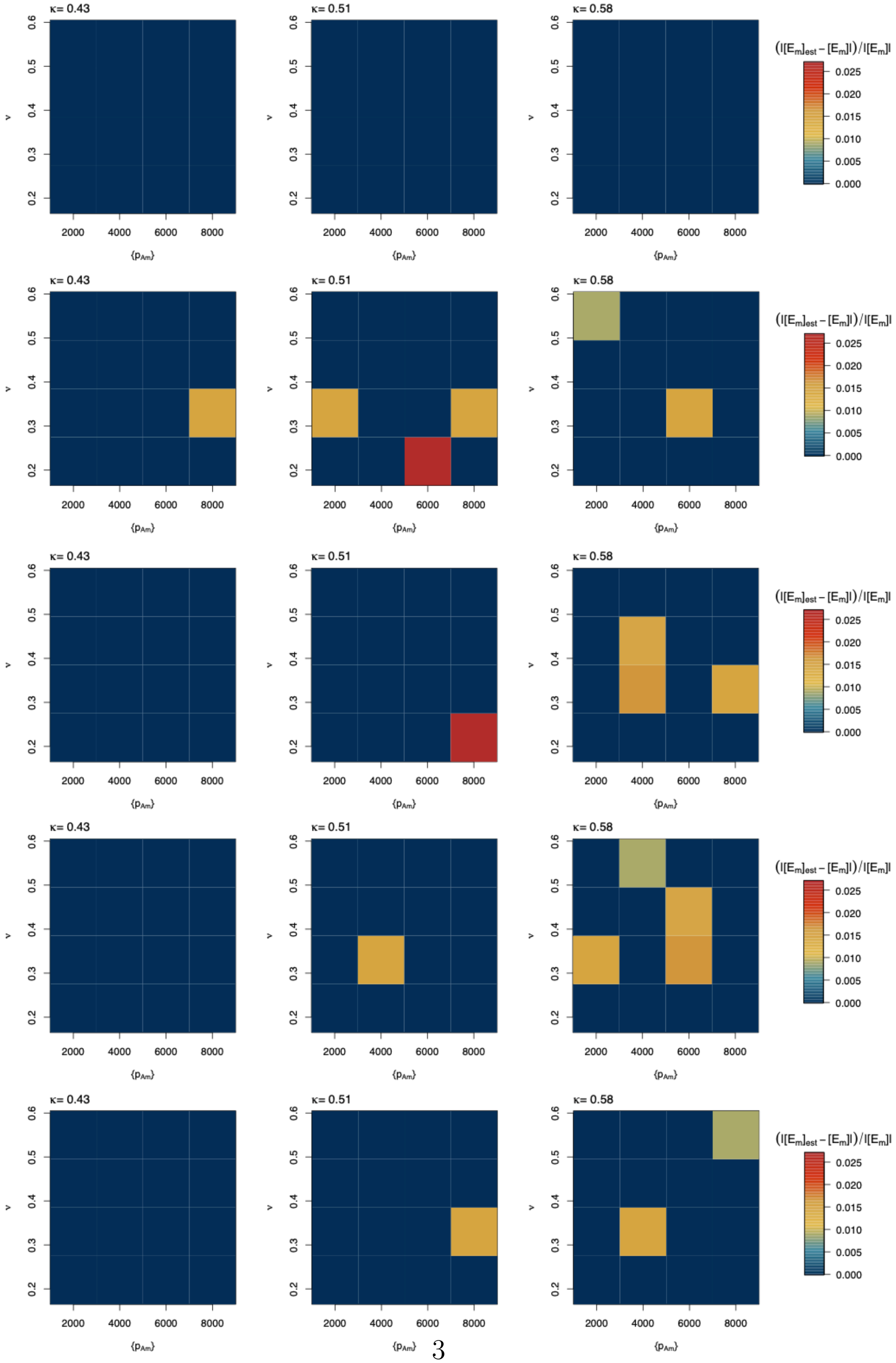

Figure 1. Relative differences between our simulations and the analytical solutions. See the figure caption in the next page.

Figure 1. (Previous page) The relative difference between the model simulations for reserve density ($[E_m]_{est}$) and the analytical solutions ($[E_m]$, equation 5) at a constant resource availability are close to zero. The rows correspond to the functional response at the five levels considered in descending order, i.e., from $f = 1$ to $f = 0.2$. The heatmap colour indicates the relative error between the numerical and the analytical solutions.

3. Effect of interspecific differences

When the resource is constant, a decreasing level minimizes the consequences of interspecific differences in reserve energy and structural volume (Figs. 2 and 3), which are directly reflected in the individual's biomass (Fig. 4 in the main text). The effect is the opposite for the development time (Fig. 4), where the interspecific differences become greater as the resource decreases. However, the differences in time to reach puberty are small, ranging from 2 to 4 days; and are likely not significant.

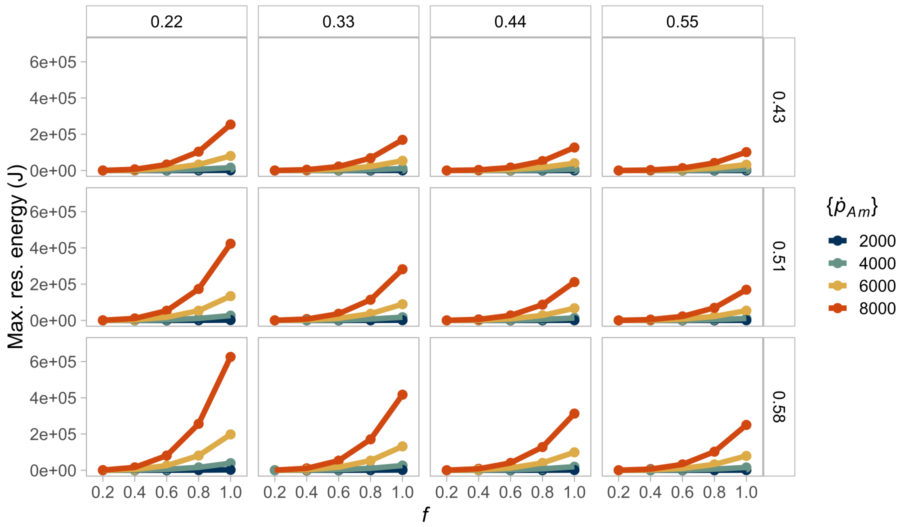

Figure 2. A constant, decreasing resource reduces interspecific differences in reserve energy. The largest reserve is attained when individuals combine high assimilation with low energy conductance. The columns indicate the value of energy conductance, while the rows represent the fraction of energy allocated to soma. Point and line colours indicate the maximum specific assimilation rate value. Points and lines of the same colour in each box (equivalent to a parameter combination) represent the same species at different food conditions.

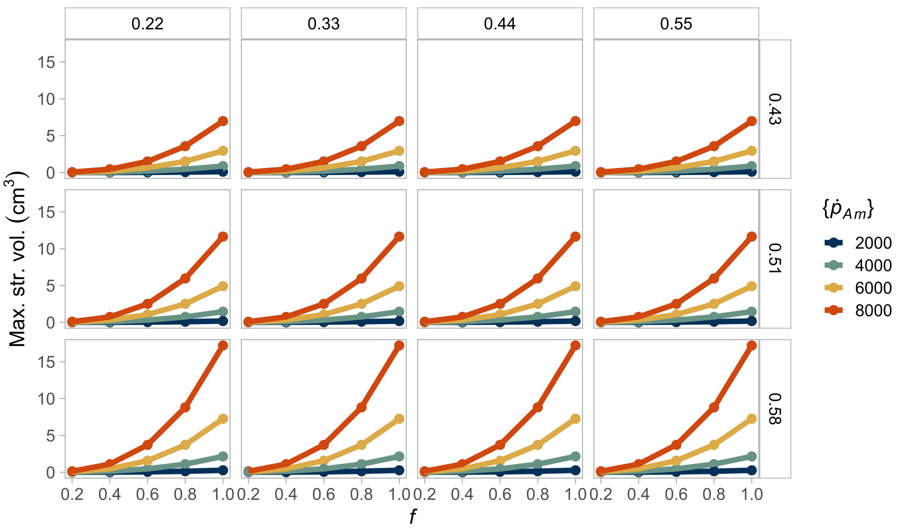

Figure 3. A constant, decreasing resource reduces interspecific differences in structural volume. The largest volume is attained when individuals combine high assimilation with low energy conductance. The columns indicate the value of energy conductance, while the rows represent the fraction of energy allocated to soma. Point and line colours indicate the maximum specific assimilation rate value. Points and lines of the same colour in each box (equivalent to a parameter combination) represent the same species at different food conditions.

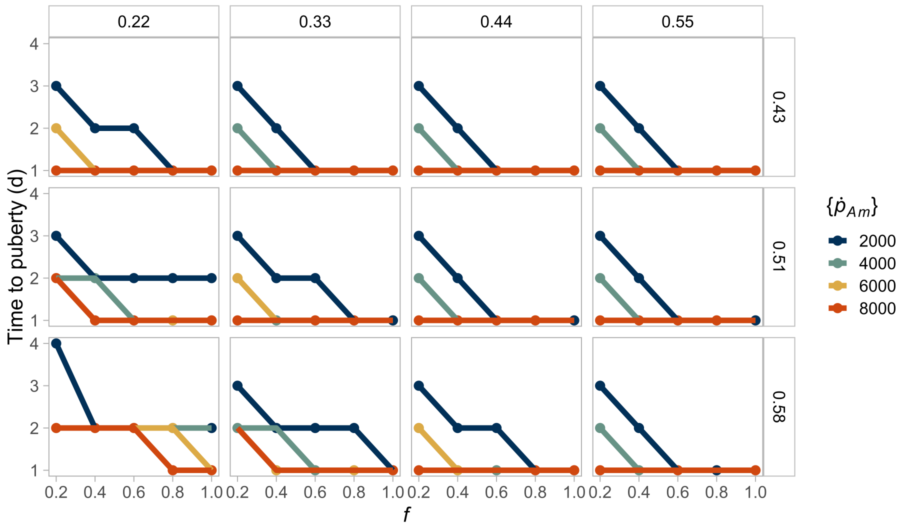

Figure 4. Interspecific differences in the time to reach puberty are amplified when a constant resource decreases. Individuals have faster developing times when the resource is non-limiting, and they combine high assimilation with high energy conductance. The columns indicate the value of energy conductance, while the rows represent the fraction of energy allocated to soma. Point and line colours indicate the maximum specific assimilation rate value. Points and lines of the same colour in each box (equivalent to a parameter combination) represent the same species at different food conditions.

The relative differences across species are nearly constant at any constant resource level for reserve energy (Fig. 5) and are uniform for structural volume (Fig. 6). The small relative differences show that the resource has mainly a scaling effect on both variables. The relative differences in developmental time are not constant but remain small and likely not significant (Fig. 7).

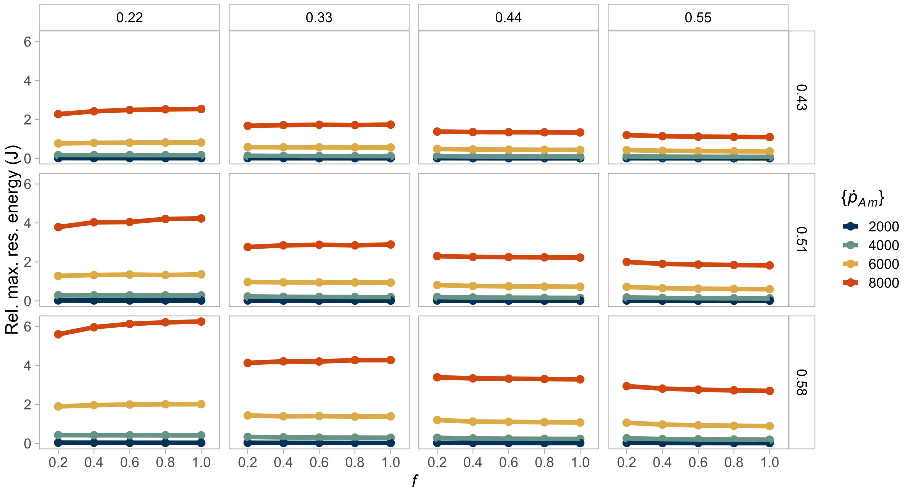

Figure 5. The resource scales the interspecific differences in reserve energy. Hence, differences between different constant food levels for the same species are small. The columns indicate the value of energy conductance, while the rows represent the fraction of energy allocated to soma. Point and line colours indicate the maximum specific assimilation rate value. Points and lines of the same colour in each box (equivalent to a parameter combination) represent the same species at different food conditions.

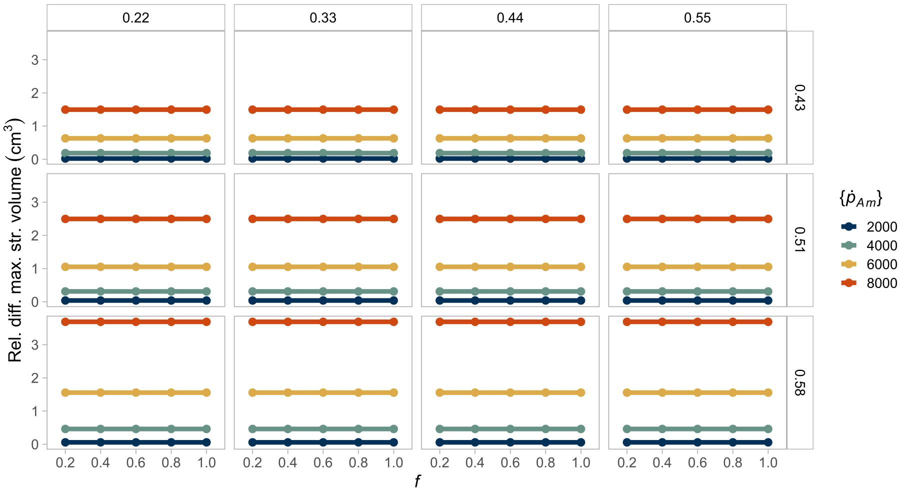

Figure 6. The resource scales the interspecific differences in structural volume. Hence, there are no relative differences between different constant food levels for the same species. The columns indicate the value of energy conductance, while the rows represent the fraction of energy allocated to soma. Point and line colours indicate the maximum specific assimilation rate value. Points and lines of the same colour in each box (equivalent to a parameter combination) represent the same species at different food conditions.

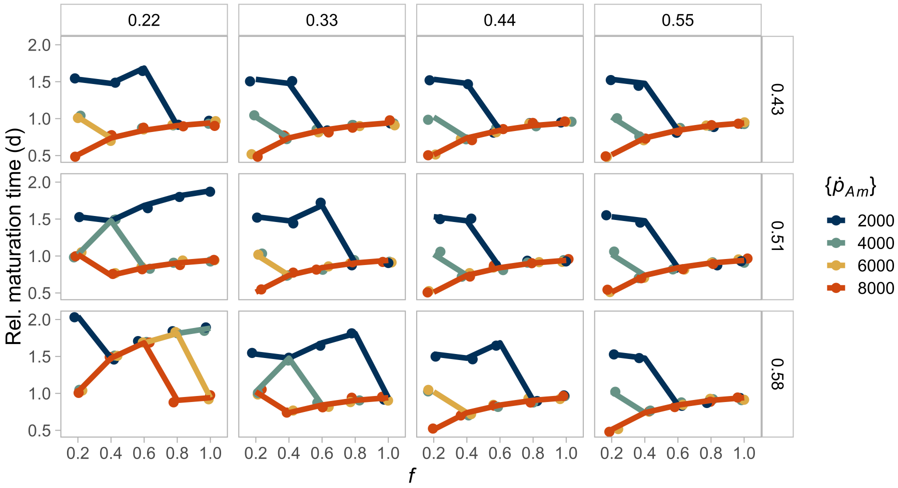

Figure 7. The relative differences between constant food levels in maturation time are small and likely not significant. The columns indicate the value of energy conductance, while the rows represent the fraction of energy allocated to soma. Point and line colours indicate the maximum specific assimilation rate value. Points and lines of the same colour in each box (equivalent to a parameter combination) represent the same species at different food conditions.

4. Effect of resource variability

A seasonal resource with a greater average amplifies the consequences of interspecific differences in biomass, reproductive output, reserve energy, and structural volume (Figs. 8 to 11, respectively). On the contrary, the interspecific differences in development time become greater as the mean resource decreases (Fig. 12). These differences in time to reach puberty are small and likely not significant. However, they depend on the initial food level or, equivalently, the birth time relative to the resource cycle.

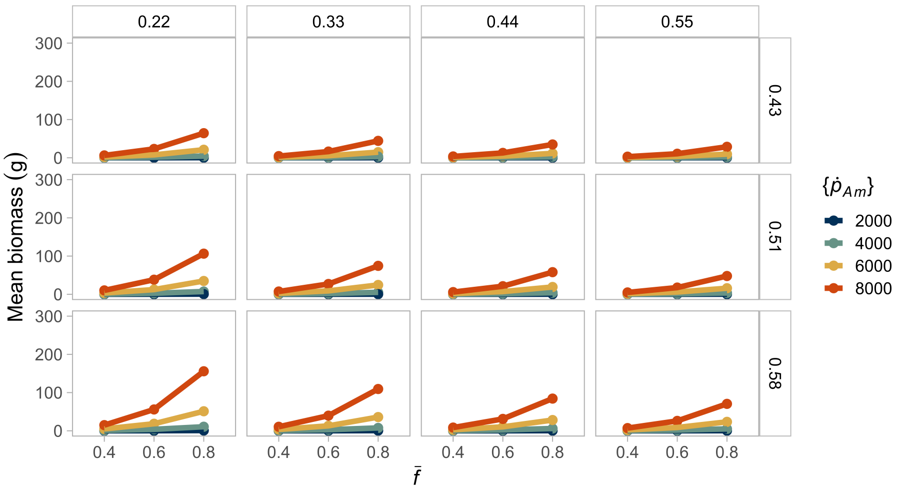

Figure 8. A seasonally varying resource with a lower average reduces interspecific differences in mean biomass, regardless of the initial food level. The largest biomass is attained when individuals combine high assimilation with low energy conductance. The columns indicate the value of energy conductance, while the rows represent the fraction of energy allocated to soma. Point and line colours indicate the maximum specific assimilation rate value. Points and lines of the same colour in each box (equivalent to a parameter combination) represent the same species at different food conditions. The value of $\bar{f}$ indicates the average resource level for each simulation. The initial resource level was set to the highest availability in each case.

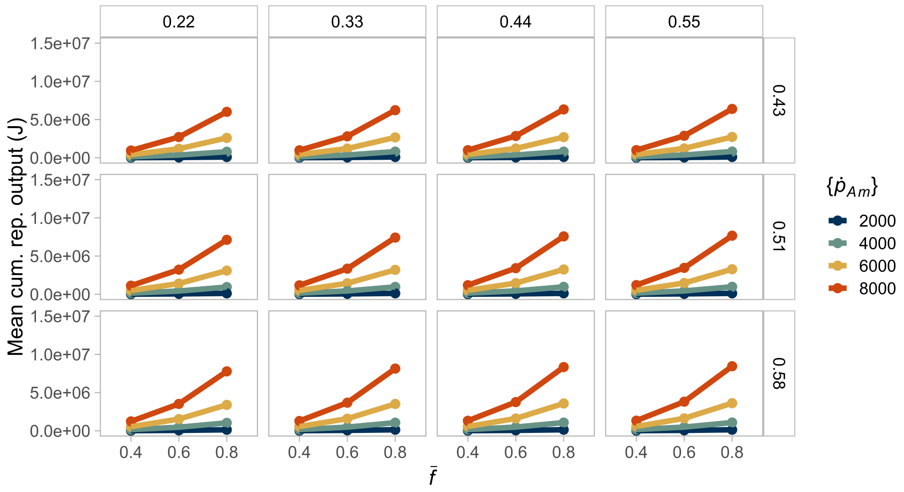

Figure 9. A seasonal and, on average scarcer resource reduces interspecific variability in mean cumulative reproductive output, regardless of the initial resource level. Higher reproductive output is reached when the fraction of energy allocated to soma is high. The columns indicate the value of energy conductance, while the rows represent the fraction of energy allocated to soma. Point and line colours indicate the maximum specific assimilation rate value. Points and lines of the same colour in each box (equivalent to a parameter combination) represent the same species at different food conditions. The value of $\bar{f}$ indicates the average resource level for each simulation. The initial resource level was set to the highest availability in each case (results are similar for all the initial food conditions, see figures 22 to 24).

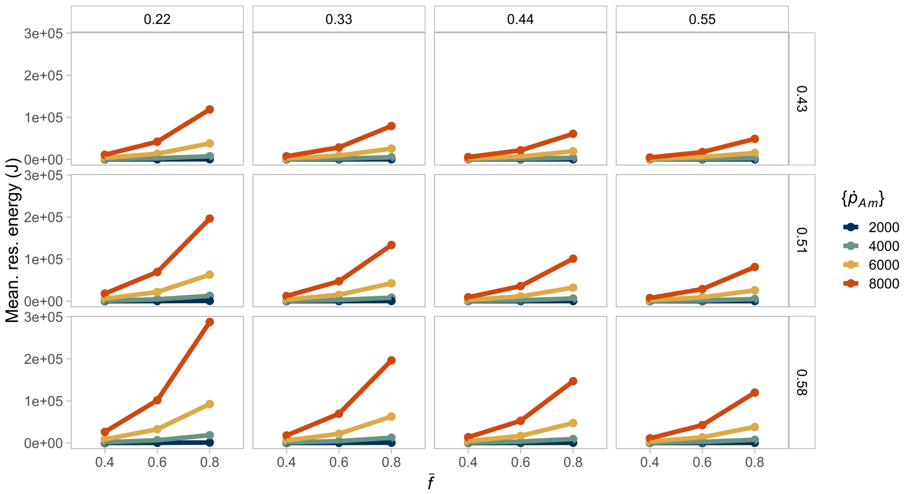

Figure 10. A seasonally varying resource with lower average reduces interspecific differences in mean reserve energy, regardless of the initial food level. The largest energy reserve is attained when individuals combine high assimilation with low energy conductance. The columns indicate the value of energy conductance, while the rows represent the fraction of energy allocated to soma. Point and line colours indicate the maximum specific assimilation rate value. Points and lines of the same colour in each box (equivalent to a parameter combination) represent the same species at different food conditions. The value of $\bar{f}$ indicates the average resource level for each simulation. The initial resource level was set to the highest availability in each case (results are similar for all the initial food conditions, see figures 16 to 18).

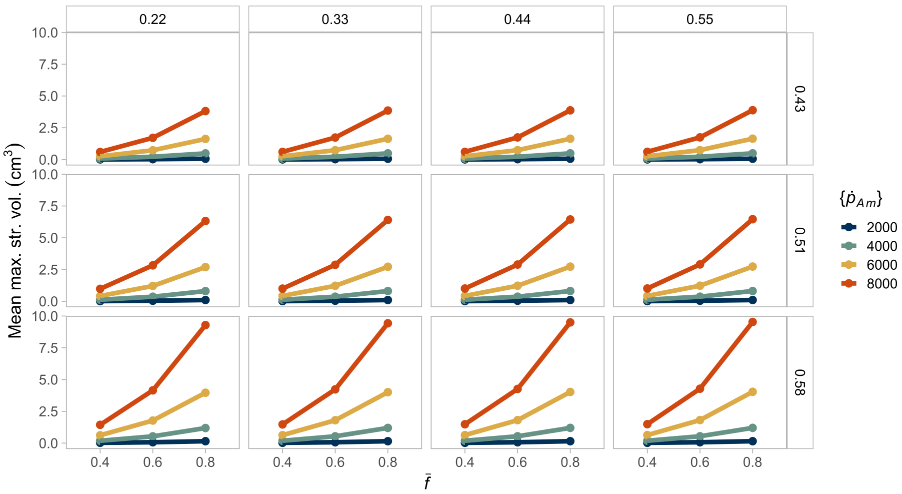

Figure 11. A seasonally varying resource with lower average reduces interspecific differences in mean structural volume, regardless of the initial food level. The largest volume is attained when individuals combine high assimilation with low energy conductance. The columns indicate the value of energy conductance, while the rows represent the fraction of energy allocated to soma. Point and line colours indicate the maximum specific assimilation rate value. Points and lines of the same colour in each box (equivalent to a parameter combination) represent the same species at different food conditions. The value of $\bar{f}$ indicates the average resource level for each simulation. The initial resource level was set to the highest availability in each case (results are similar for all the initial food conditions, see figures 19 to 21).

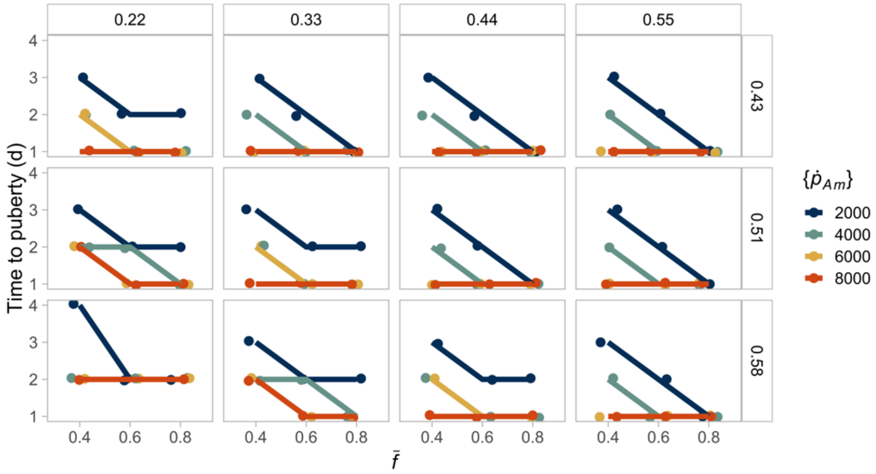

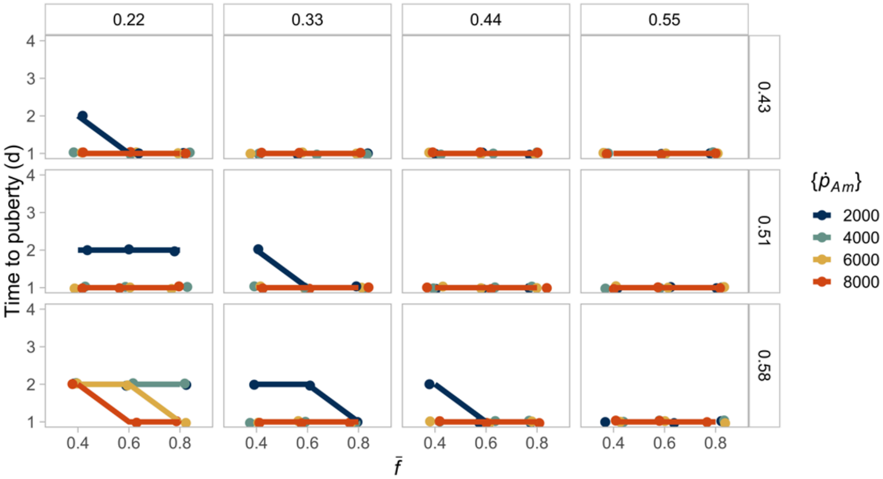

Figure 12. Interspecific differences in the time to reach puberty are amplified when the level of the resource is, on average, lower. A low initial resource produces individuals with slower development times (top) than the same individuals with maximum initial resource availability (bottom). In both scenarios, individuals develop faster when the resource is non-limiting, and they combine high assimilation with high energy conductance. The columns indicate the value of energy conductance, while the rows represent the fraction of energy allocated to soma. Point and line colours indicate the maximum specific assimilation rate value. Points and lines of the same colour in each box (equivalent to a parameter combination) represent the same species at different food conditions. The value of $\bar{f}$ indicates the average resource level for each simulation (see figures 25 to 27 for comparison among different initial resource conditions).

5. Comparison across initial resource conditions

Regardless of the initial resource level, seasonality amplifies the consequences of interspecific differences in biomass (Figs. 13 to 15), reserve energy (Figs. 16 to 18), structural volume (Figs. 19 to 21), and reproductive output (Figs. 22 to 24). The mean resource level seems to have a greater effect than the initial resource conditions on the development time (Figs. 25 to 27). However, the differences in time to reach puberty are likely not significant because their variation ranges from 2 to 4 days.

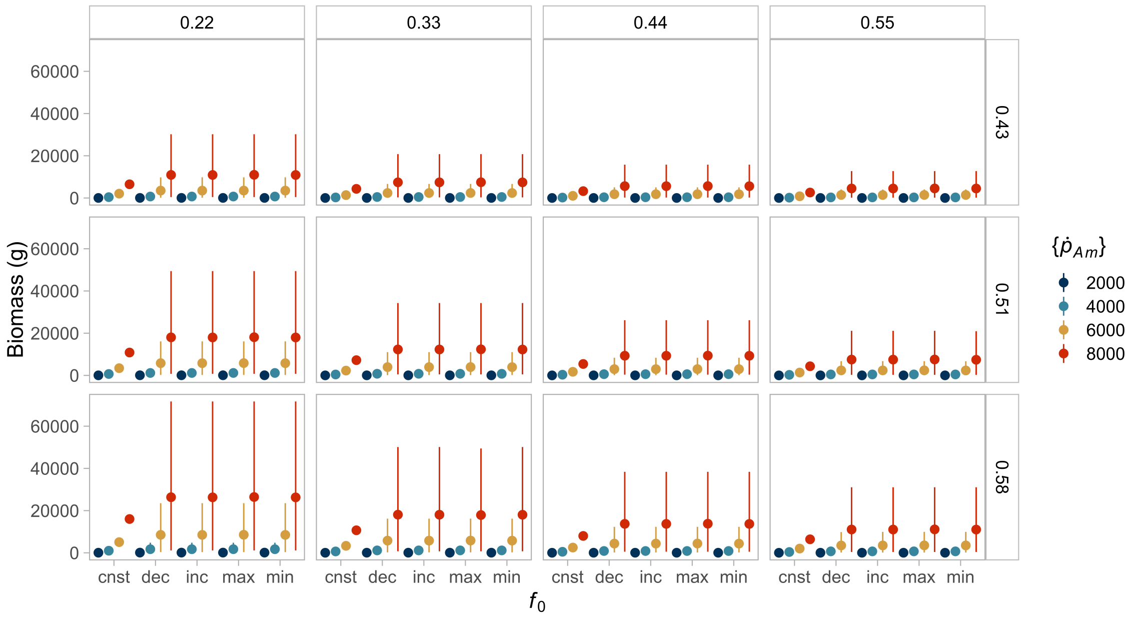

Figure 13. Individuals in a seasonal environment reach larger average biomass than the same individual in a constant environment with an equal mean resource availability, regardless of the initial resource condition f_0. Here, we compare individuals in a constant resource environment (cnst, $f = 0.4$) to those experiencing seasonality ($\bar{f} = 0.4$). We contrasted four different initial conditions for the seasonal environment, which means that individuals can be born when the resource is decreasing (dec, $f_0 = 0.3$), at the minimum level (min, $f_0 = 0.2$), increasing (inc, $f_0 = 0.3$) or at the maximum level (max, $f_0 = 0.4$). In the constant environment, points show the steady-state value reached at the end of the simulations (i.e., year three). In contrast, for the seasonal environment, points represent the average reserve energy calculated over the last two years (i.e., years one to three), and lines correspond to the minimum and maximum values. The columns indicate the value of energy conductance, while the rows represent the fraction of energy allocated to soma. Point and line colours indicate the maximum specific assimilation rate value. Points and lines of the same colour in each box (equivalent to a parameter combination) represent the same species at different food conditions.

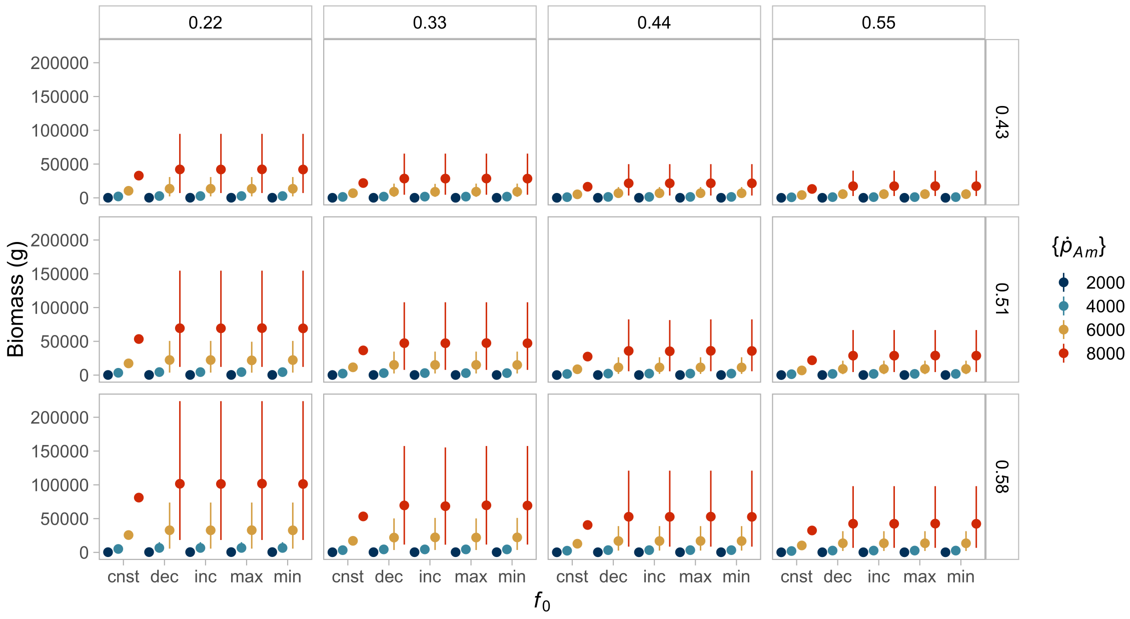

Figure 14. Individuals in a seasonal environment reach larger average biomass than the same individual in a constant environment with an equal mean resource availability, regardless of the initial resource condition f_0. Here, we compare individuals in a constant resource environment (cnst, $f = 0.6$) to those experiencing seasonality ($\bar{f} = 0.6$). We contrasted four different initial conditions for the seasonal environment, which means that individuals can be born when the resource is decreasing (dec, $f_0 = 0.5$), at the minimum level (min, $f_0 = 0.4$), increasing (inc, $f_0 = 0.5$) or at the maximum level (max, $f_0 = 0.8$). In the constant environment, points show the steady-state value reached at the end of the simulations (i.e., year three). In contrast, for the seasonal environment, points represent the average reserve energy calculated over the last two years (i.e., years one to three), and lines correspond to the minimum and maximum values. The columns indicate the value of energy conductance, while the rows represent the fraction of energy allocated to soma. Point and line colours indicate the maximum specific assimilation rate value. Points and lines of the same colour in each box (equivalent to a parameter combination) represent the same species at different food conditions.

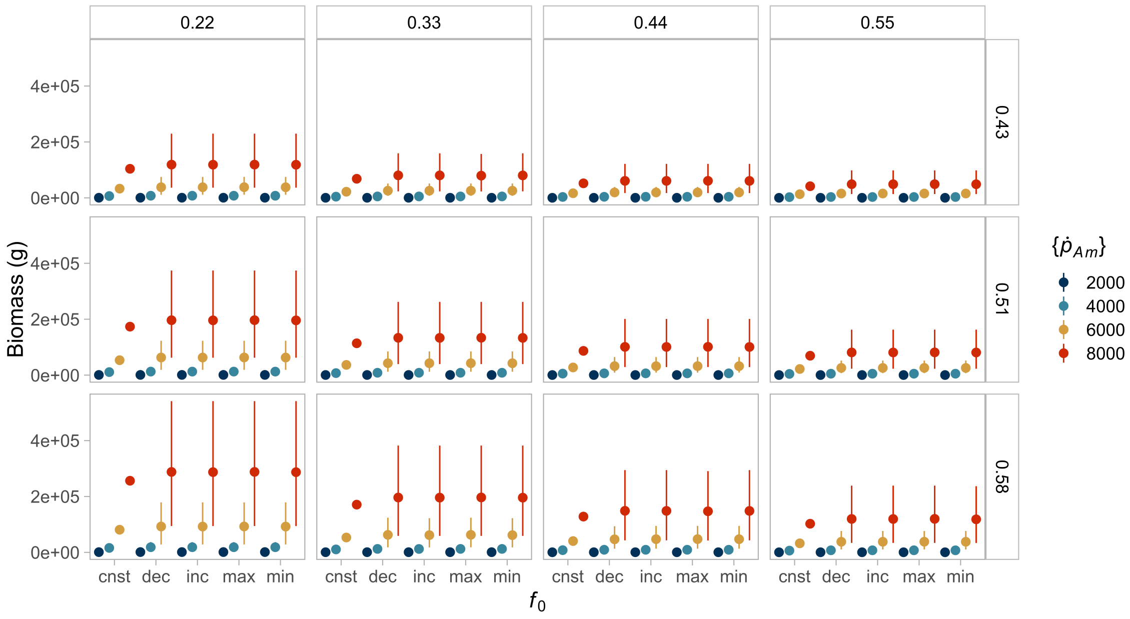

Figure 15. Individuals in a seasonal environment reach larger average biomass than the same individual in a constant environment with an equal mean resource availability, regardless of the initial resource condition f_0. Here, we compare individuals in a constant resource environment (cnst, $f = 0.8$) to those experiencing seasonality ($\bar{f} = 0.8$). We contrasted four different initial conditions for the seasonal environment, which means that individuals can be born when the resource is decreasing (dec, $f_0 = 0.7$), at the minimum level (min, $f_0 = 0.6$), increasing (inc, $f_0 = 0.7$) or at the maximum level (max, $f_0 = 1$). In the constant environment, points show the steady-state value reached at the end of the simulations (i.e., year three). In contrast, for the seasonal environment, points represent the average reserve energy calculated over the last two years (i.e., years one to three), and lines correspond to the minimum and maximum values. The columns indicate the value of energy conductance, while the rows represent the fraction of energy allocated to soma. Point and line colours indicate the maximum specific assimilation rate value. Points and lines of the same colour in each box (equivalent to a parameter combination) represent the same species at different food conditions.

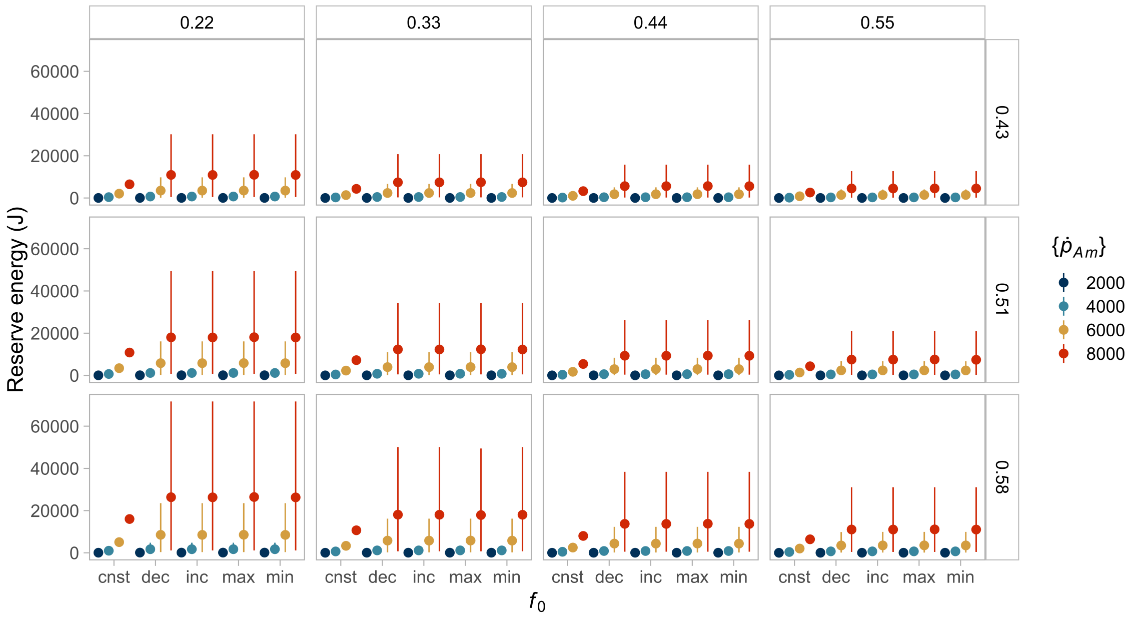

Figure 16. Individuals in a seasonal environment reach larger average reserve energy than the same individual in a constant environment with an equal mean resource availability, regardless of the initial resource condition f_0. Here, we compare individuals in a constant resource environment (cnst, $f = 0.4$) to those experiencing seasonality ($\bar{f} = 0.4$). We contrasted four different initial conditions for the seasonal environment, which means that individuals can be born when the resource is decreasing (dec, $f_0 = 0.3$), at the minimum level (min, $f_0 = 0.2$), increasing (inc, $f_0 = 0.3$) or at the maximum level (max, $f_0 = 0.4$). In the constant environment, points show the steady-state value reached at the end of the simulations (i.e., year three). In contrast, for the seasonal environment, points represent the average reserve energy calculated over the last two years (i.e., years one to three), and lines correspond to the minimum and maximum values. The columns indicate the value of energy conductance, while the rows represent the fraction of energy allocated to soma. Point and line colours indicate the maximum specific assimilation rate value. Points and lines of the same colour in each box (equivalent to a parameter combination) represent the same species at different food conditions.

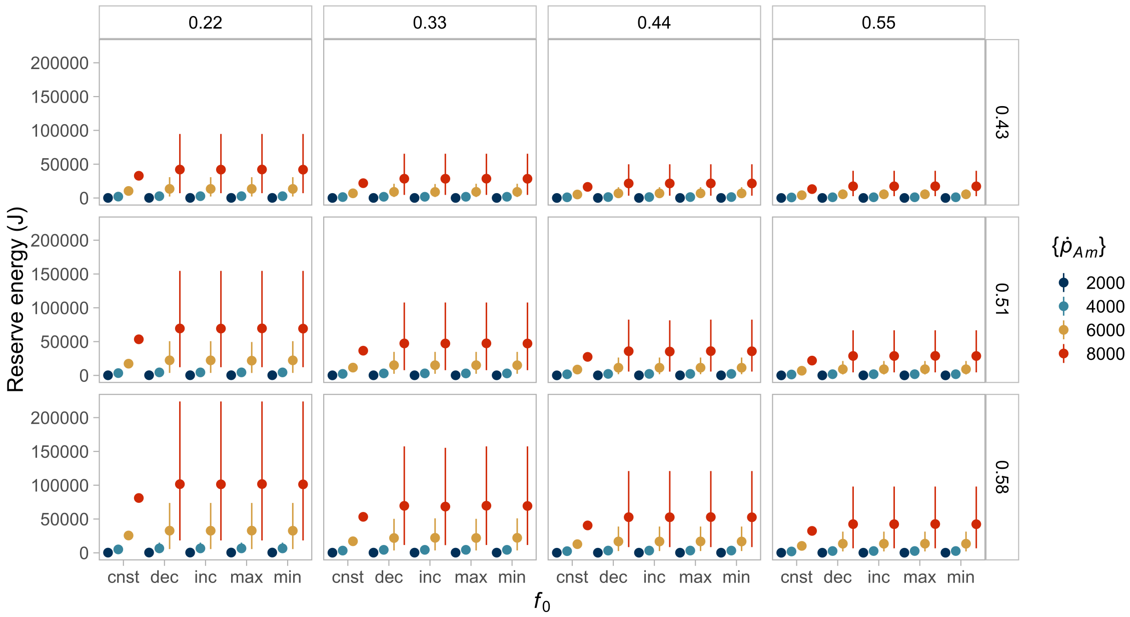

Figure 17. Individuals in a seasonal environment reach larger average reserve energy than the same individual in a constant environment with an equal mean resource availability, regardless of the initial resource condition f_0. Here, we compare individuals in a constant resource environment (cnst, $f = 0.6$) to those experiencing seasonality ($\bar{f} = 0.6$). We contrasted four different initial conditions for the seasonal environment, which means that individuals can be born when the resource is decreasing (dec, $f_0 = 0.5$), at the minimum level (min, $f_0 = 0.4$), increasing (inc, $f_0 = 0.5$) or at the maximum level (max, $f_0 = 0.8$). In the constant environment, points show the steady-state value reached at the end of the simulations (i.e., year three). In contrast, for the seasonal environment, points represent the average reserve energy calculated over the last two years (i.e., years one to three), and lines correspond to the minimum and maximum values. The columns indicate the value of energy conductance, while the rows represent the fraction of energy allocated to soma. Point and line colours indicate the maximum specific assimilation rate value. Points and lines of the same colour in each box (equivalent to a parameter combination) represent the same species at different food conditions.

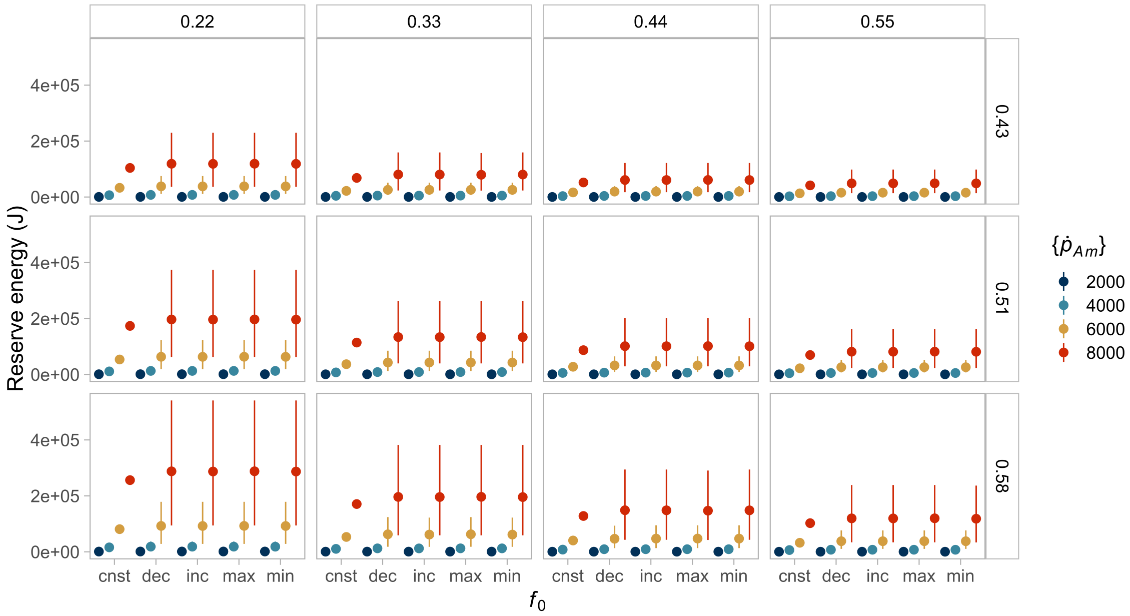

Figure 18. Individuals in a seasonal environment reach larger average reserve energy than the same individual in a constant environment with an equal mean resource availability, regardless of the initial resource condition f_0. Here, we compare individuals in a constant resource environment (cnst, $f = 0.8$) to those experiencing seasonality ($\bar{f} = 0.8$). We contrasted four different initial conditions for the seasonal environment, which means that individuals can be born when the resource is decreasing (dec, $f_0 = 0.7$), at the minimum level (min, $f_0 = 0.6$), increasing (inc, $f_0 = 0.7$) or at the maximum level (max, $f_0 = 1$). In the constant environment, points show the steady-state value reached at the end of the simulations (i.e., year three). In contrast, for the seasonal environment, points represent the average reserve energy calculated over the last two years (i.e., years one to three), and lines correspond to the minimum and maximum values. The columns indicate the value of energy conductance, while the rows represent the fraction of energy allocated to soma. Point and line colours indicate the maximum specific assimilation rate value. Points and lines of the same colour in each box (equivalent to a parameter combination) represent the same species at different food conditions.

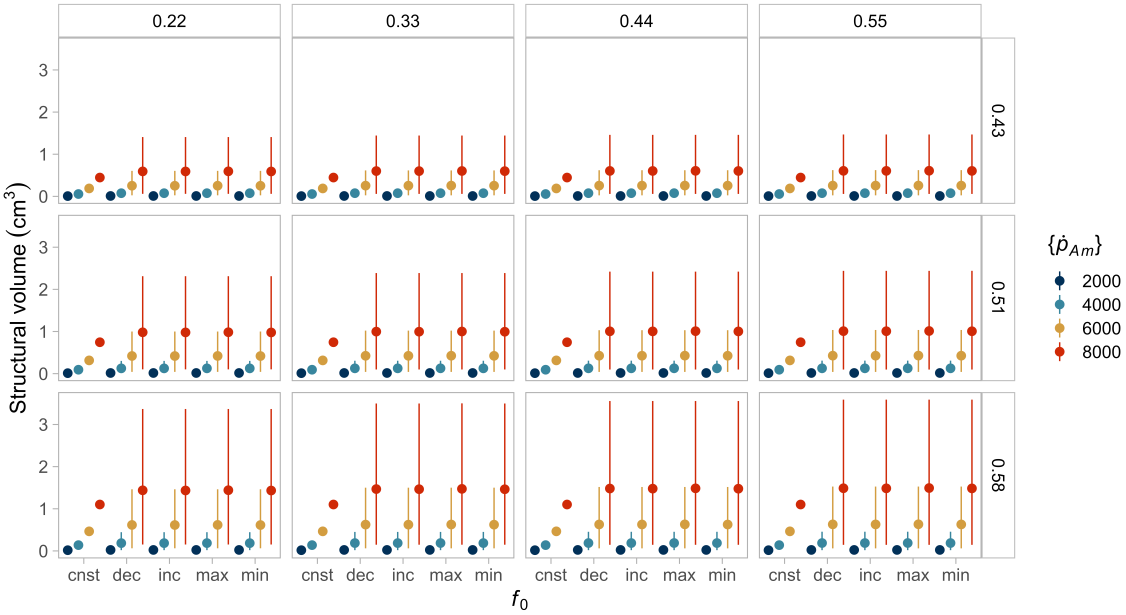

Figure 19. Individuals in a seasonal environment reach greater average structural volume than the same individual in a constant environment with an equal mean resource availability, regardless of the initial resource condition f_0. Here, we compare individuals in a constant resource environment (cnst, $f = 0.4$) to those experiencing seasonality ($\bar{f} = 0.4$). We contrasted four different initial conditions for the seasonal environment, which means that individuals can be born when the resource is decreasing (dec, $f_0 = 0.3$), at the minimum level (min, $f_0 = 0.2$), increasing (inc, $f_0 = 0.3$) or at the maximum level (max, $f_0 = 0.4$). In the constant environment, points show the steady-state value reached at the end of the simulations (i.e., year three). In contrast, for the seasonal environment, points represent the average structural volume calculated over the last two years (i.e., years one to three), and lines correspond to the minimum and maximum values. The columns indicate the value of energy conductance, while the rows represent the fraction of energy allocated to soma. Point and line colours indicate the maximum specific assimilation rate value. Points and lines of the same colour in each box (equivalent to a parameter combination) represent the same species at different food conditions.

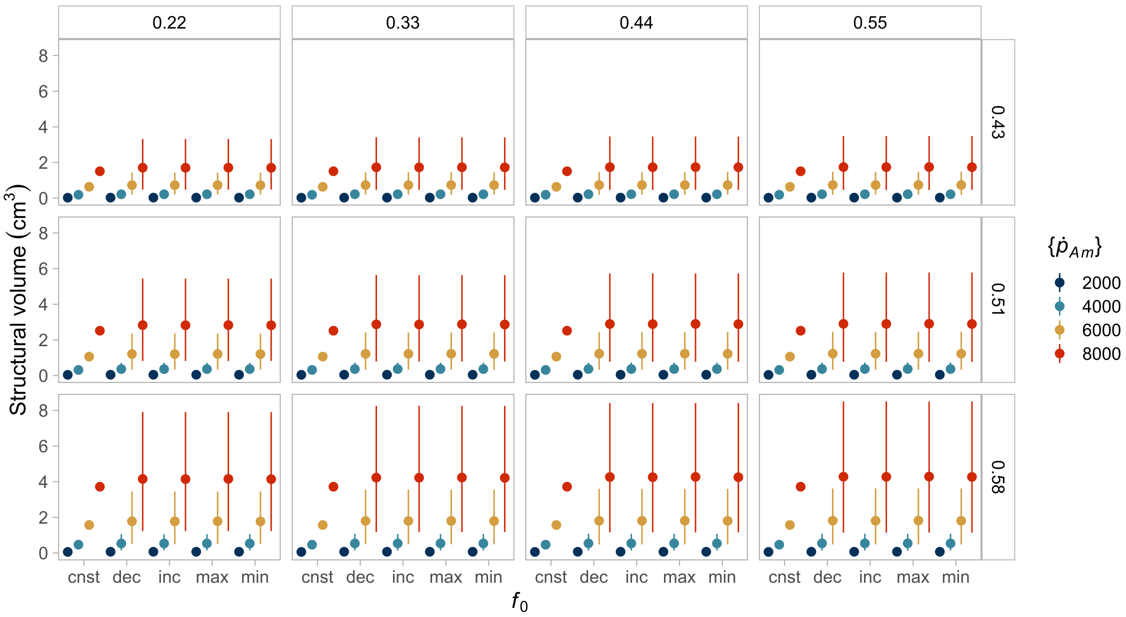

Figure 20. Individuals in a seasonal environment reach greater average structural volume than the same individual in a constant environment with an equal mean resource availability, regardless of the initial resource condition f_0. Here, we compare individuals in a constant resource environment (cnst, $f = 0.6$) to those experiencing seasonality ($\bar{f} = 0.6$). We contrasted four different initial conditions for the seasonal environment, which means that individuals can be born when the resource is decreasing (dec, $f_0 = 0.5$), at the minimum level (min, $f_0 = 0.4$), increasing (inc, $f_0 = 0.5$) or at the maximum level (max, $f_0 = 0.8$). In the constant environment, points show the steady-state value reached at the end of the simulations (i.e., year three). In contrast, for the seasonal environment, points represent the average structural volume calculated over the last two years (i.e., years one to three), and lines correspond to the minimum and maximum values. The columns indicate the value of energy conductance, while the rows represent the fraction of energy allocated to soma. Point and line colours indicate the maximum specific assimilation rate value. Points and lines of the same colour in each box (equivalent to a parameter combination) represent the same species at different food conditions.

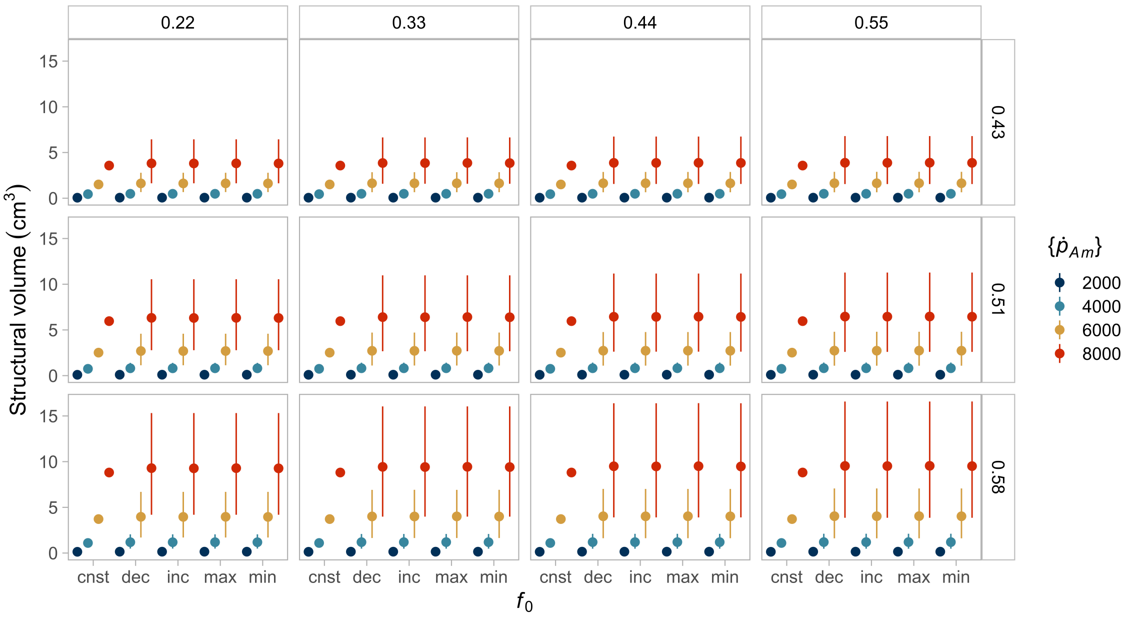

Figure 21. Individuals in a seasonal environment reach greater average structural volume than the same individual in a constant environment with an equal mean resource availability, regardless of the initial resource condition f_0. Here, we compare individuals in a constant resource environment (cnst, $f = 0.8$) to those experiencing seasonality ($\bar{f} = 0.8$). We contrasted four different initial conditions for the seasonal environment, which means that individuals can be born when the resource is decreasing (dec, $f_0 = 0.7$), at the minimum level (min, $f_0 = 0.6$), increasing (inc, $f_0 = 0.7$) or at the maximum level (max, $f_0 = 1$). In the constant environment, points show the steady-state value reached at the end of the simulations (i.e., year three). In contrast, for the seasonal environment, points represent the average structural volume calculated over the last two years (i.e., years one to three), and lines correspond to the minimum and maximum values. The columns indicate the value of energy conductance, while the rows represent the fraction of energy allocated to soma. Point and line colours indicate the maximum specific assimilation rate value. Points and lines of the same colour in each box (equivalent to a parameter combination) represent the same species at different food conditions.

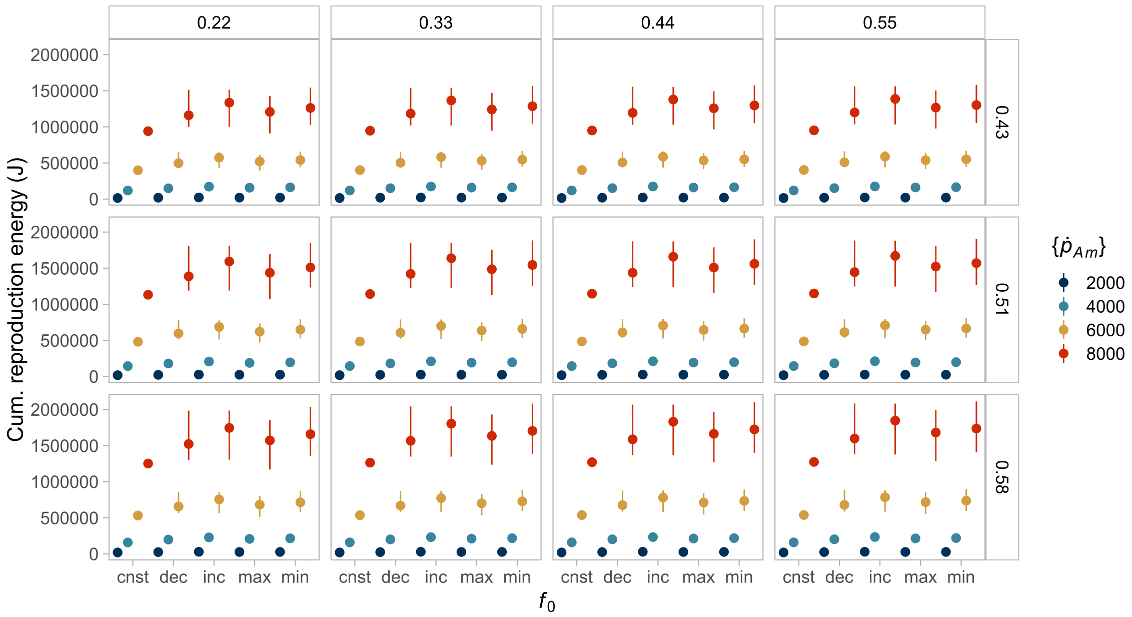

Figure 22. Individuals in a seasonal environment reach greater average cumulative reproduction energy than the same individual in a constant environment with an equal mean resource availability, regardless of the initial resource condition f_0. Here, we compare individuals in a constant resource environment (cnst, $f = 0.4$) to those experiencing seasonality ($\bar{f} = 0.4$). We contrasted four different initial conditions for the seasonal environment, which means that individuals can be born when the resource is decreasing (dec, $f_0 = 0.3$), at the minimum level (min, $f_0 = 0.2$), increasing (inc, $f_0 = 0.3$) or at the maximum level (max, $f_0 = 0.4$). In the constant environment, points show the steady-state value reached at the end of the simulations (i.e., year three). In contrast, for the seasonal environment, points represent the average structural volume calculated over the last two years (i.e., years one to three), and lines correspond to the minimum and maximum values. The columns indicate the value of energy conductance, while the rows represent the fraction of energy allocated to soma. Point and line colours indicate the maximum specific assimilation rate value. Points and lines of the same colour in each box (equivalent to a parameter combination) represent the same species at different food conditions.

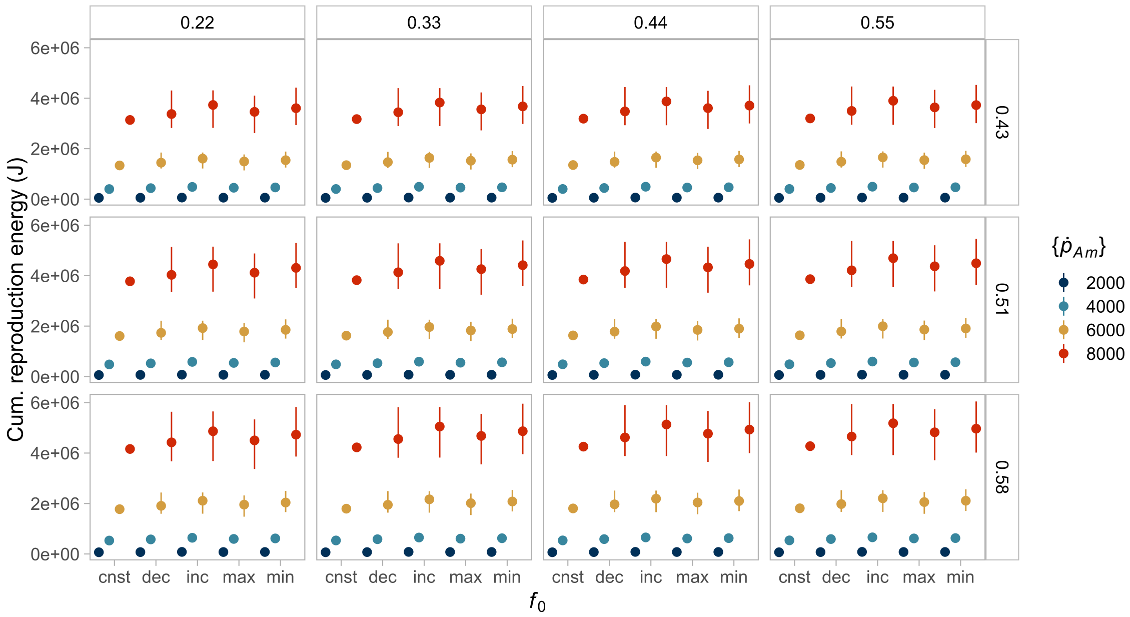

Figure 23. Individuals in a seasonal environment reach greater average cumulative reproduction energy than the same individual in a constant environment with an equal mean resource availability, regardless of the initial resource condition f_0. Here, we compare individuals in a constant resource environment (cnst, $f = 0.6$) to those experiencing seasonality ($\bar{f} = 0.6$). We contrasted four different initial conditions for the seasonal environment, which means that individuals can be born when the resource is decreasing (dec, $f_0 = 0.5$), at the minimum level (min, $f_0 = 0.4$), increasing (inc, $f_0 = 0.5$) or at the maximum level (max, $f_0 = 0.8$). In the constant environment, points show the steady-state value reached at the end of the simulations (i.e., year three). In contrast, for the seasonal environment, points represent the average structural volume calculated over the last two years (i.e., years one to three), and lines correspond to the minimum and maximum values. The columns indicate the value of energy conductance, while the rows represent the fraction of energy allocated to soma. Point and line colours indicate the maximum specific assimilation rate value. Points and lines of the same colour in each box (equivalent to a parameter combination) represent the same species at different food conditions.

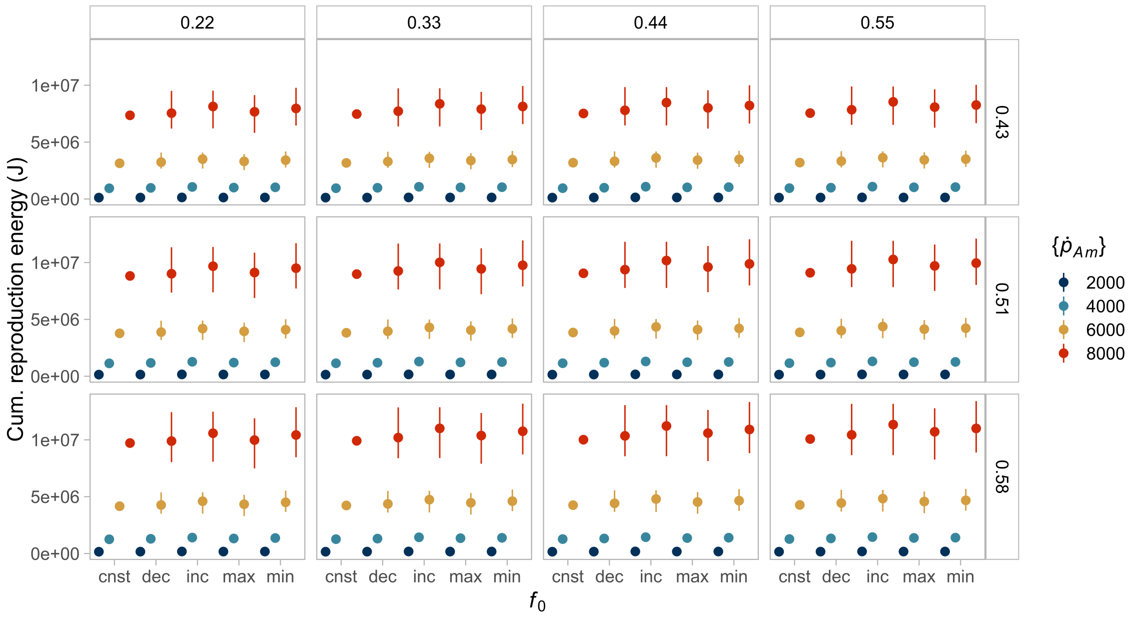

Figure 24. Individuals in a seasonal environment reach greater average cumulative reproduction energy than the same individual in a constant environment with an equal mean resource availability, regardless of the initial resource condition f_0. Here, we compare individuals in a constant resource environment (cnst, $f = 0.8$) to those experiencing seasonality ($\bar{f} = 0.8$). We contrasted four different initial conditions for the seasonal environment, which means that individuals can be born when the resource is decreasing (dec, $f_0 = 0.7$), at the minimum level (min, $f_0 = 0.6$), increasing (inc, $f_0 = 0.7$) or at the maximum level (max, $f_0 = 1$). In the constant environment, points show the steady-state value reached at the end of the simulations (i.e., year three). In contrast, for the seasonal environment, points represent the average structural volume calculated over the last two years (i.e., years one to three), and lines correspond to the minimum and maximum values. The columns indicate the value of energy conductance, while the rows represent the fraction of energy allocated to soma. Point and line colours indicate the maximum specific assimilation rate value. Points and lines of the same colour in each box (equivalent to a parameter combination) represent the same species at different food conditions.

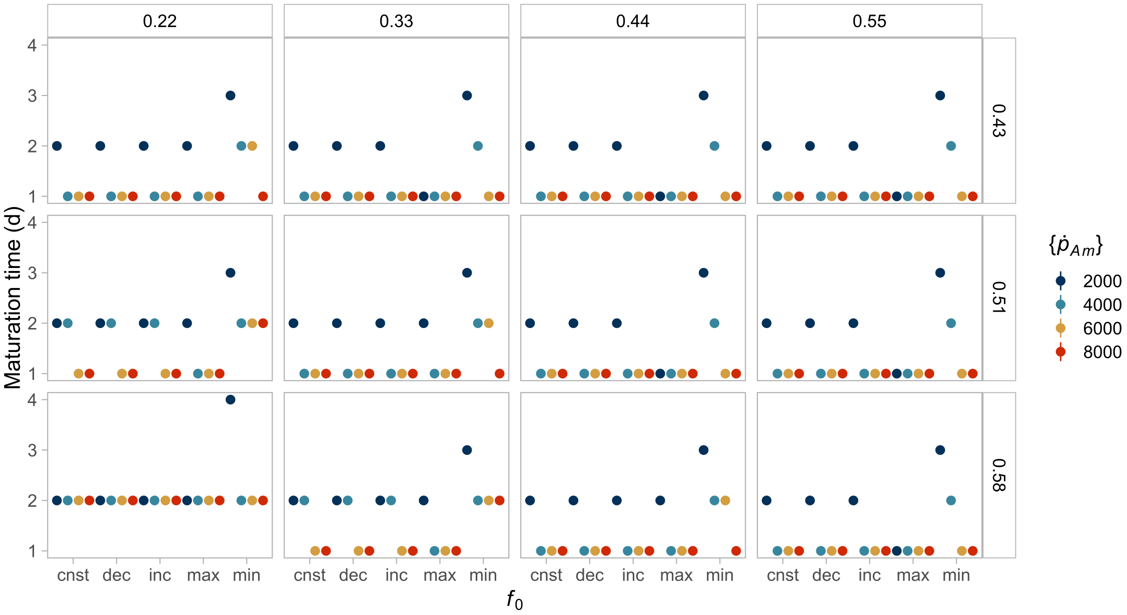

Figure 25. Interspecific differences in the time to reach puberty between individuals in a seasonal environment and the same individual in a constant environment with an equal mean resource availability do not seem to be dependent on the initial resource condition, f_0. Here, we compare individuals in a constant resource environment (cnst, $f = 0.4$) to those experiencing seasonality ($\bar{f} = 0.4$). We contrasted four different initial conditions for the seasonal environment, which means that individuals can be born when the resource is decreasing (dec, $f_0 = 0.3$), at the minimum level (min, $f_0 = 0.2$), increasing (inc, $f_0 = 0.3$) or at the maximum level (max, $f_0 = 0.6$). In all environments, points show the numbers of days required to reach the puberty threshold (E_H^p). The columns indicate the value of energy conductance, while the rows represent the fraction of energy allocated to soma. Point colours indicate the maximum specific assimilation rate value. Points of the same colour in each box (equivalent to a parameter combination) represent the same species at different food conditions.

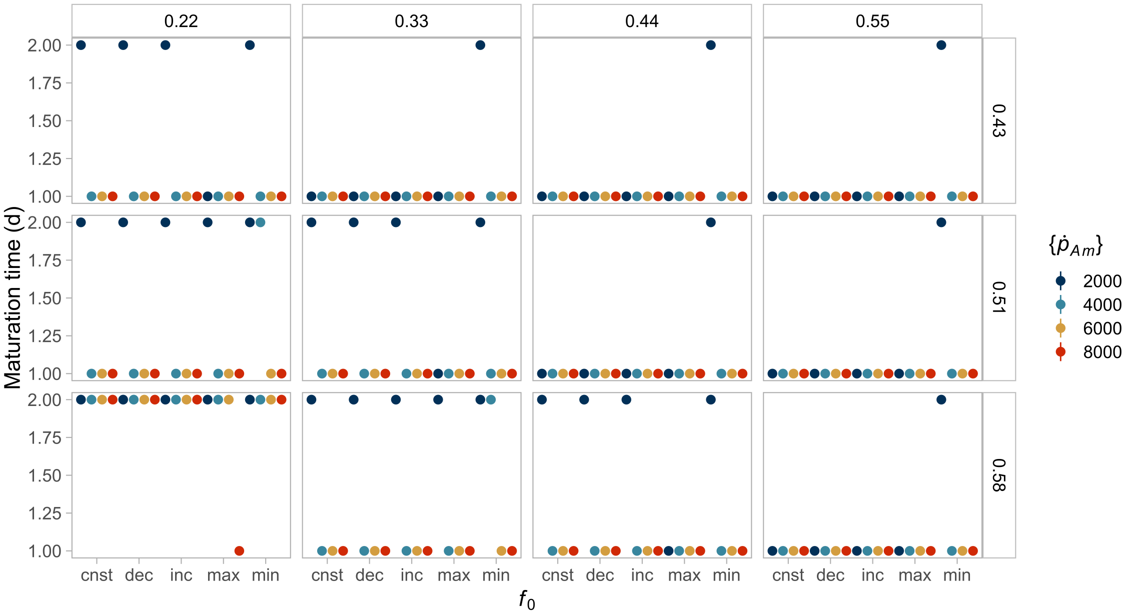

Figure 26. Interspecific differences in the time to reach puberty between individuals in a seasonal environment and the same individual in a constant environment with an equal mean resource availability do not seem to be dependent on the initial resource condition, f_0. Here, we compare individuals in a constant resource environment (cnst, $f = 0.6$) to those experiencing seasonality ($\bar{f} = 0.6$). We contrasted four different initial conditions for the seasonal environment, which means that individuals can be born when the resource is decreasing (dec, $f_0 = 0.5$), at the minimum level (min, $f_0 = 0.4$), increasing (inc, $f_0 = 0.5$) or at the maximum level (max, $f_0 = 0.8$). In all environments, points show the numbers of days required to reach the puberty threshold (E_H^p). The columns indicate the value of energy conductance, while the rows represent the fraction of energy allocated to soma. Point colours indicate the maximum specific assimilation rate value. Points of the same colour in each box (equivalent to a parameter combination) represent the same species at different food conditions.

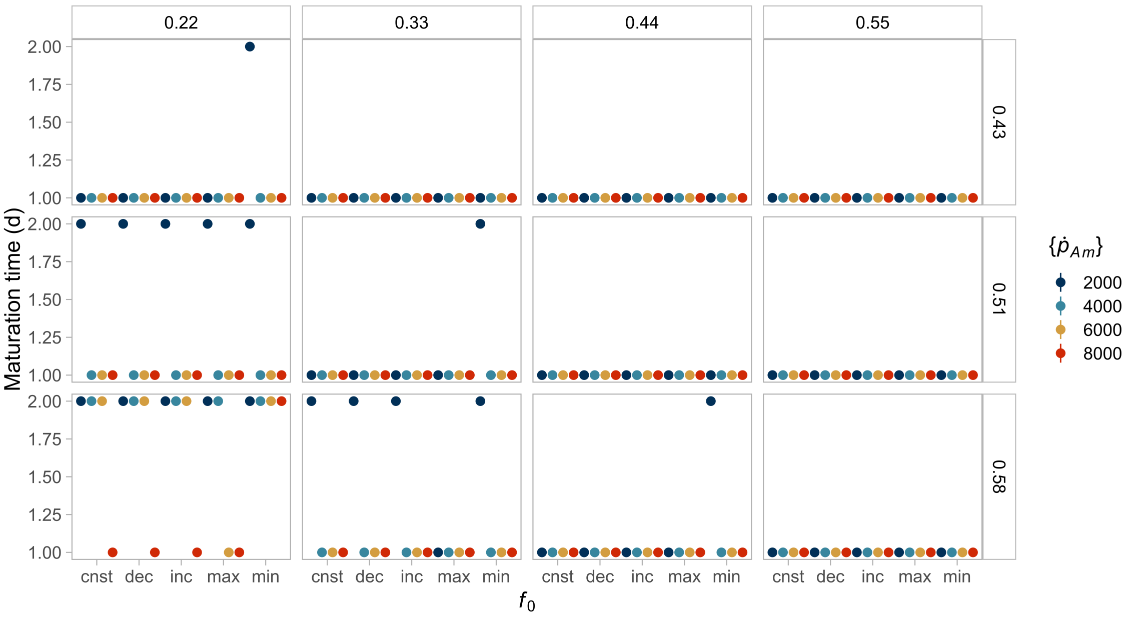

Figure 27. Interspecific differences in the time to reach puberty between individuals in a seasonal environment and the same individual in a constant environment with an equal mean resource availability do not seem to be dependent on the initial resource condition, f_0. Here, we compare individuals in a constant resource environment (cnst, $f = 0.8$) to those experiencing seasonality ($\bar{f} = 0.8$). We contrasted four different initial conditions for the seasonal environment, which means that individuals can be born when the resource is decreasing (dec, $f_0 = 0.7$), at the minimum level (min, $f_0 = 0.6$), increasing (inc, $f_0 = 0.7$) or at the maximum level (max, $f_0 = 1$). In all environments, points show the numbers of days required to reach the puberty threshold (E_H^p). The columns indicate the value of energy conductance, while the rows represent the fraction of energy allocated to soma. Point colours indicate the maximum specific assimilation rate value. Points of the same colour in each box (equivalent to a parameter combination) represent the same species at different food conditions.

6. Comparing temperate and tropical species: an example

We used the fan-tailed gerygone, *Gerygone flavolateralis*, and the grey warbler, *G. igata*, to compare the traits exhibited by related species in contrasting environments. We chose these species because they were originally within our parameter space. However, we included more data and reestimated their parameters to improve the accuracy of the predictions (Tab. 2).

Table 2. Parameter values for the two species used to compare tropical versus temperate species.

Species	Common name	κ	$\{\dot{p}_{Am}\}$	$\dot{v}$	$[\dot{p}_M]$	$[E_G]$	E_H^b	E_H^p
Gerygone flavolateralis	Fan-tailed gerygone	0.998	4692.6	0.08	5084.7	7338	7.647	453.1
Gerygone igata	Grey warbler	0.983	4878.1	0.05	6045.1	7351	71.74	7880
